# Purification and Partial Characterization of a Novel Bacteriocin Synthesized by *Lactobacillus paracasei* HD1-7 Isolated from Chinese Sauerkraut Juice

**DOI:** 10.1038/srep19366

**Published:** 2016-01-14

**Authors:** Jingping Ge, Yanyang Sun, Xing Xin, Ying Wang, Wenxiang Ping

**Affiliations:** 1 Heilongjiang University, Harbin, Heilongjiang, China

## Abstract

Bacteriocins have antimicrobial activities against food-spoiling bacteria and food-borne pathogens. Paracin 1.7, a bacteriocin synthesized by *Lactobacillus paracasei* HD1-7 isolated from Chinese sauerkraut juice, was studied. Following partial purification with ammonium sulfate precipitation, CM Sepharose Fast Flow, and Sephadex G-10 chromatography, the molecular weight of Paracin 1.7 was about 10 kDa based on Tricine-SDS-PAGE results. A 2.87 fold purified bacteriocin was produced, reaching a final yield of 39.93% and the specific activity of 1.56 × 10^3^ AU/mg. The N-terminal amino acid sequence of Paracin 1.7 was VSNTFFA, and the LC/LTQ results revealed that the N-terminal amino acid sequence was similar to that of ABC-type oligopeptide transport system protein and N-acetylmuramoyl-L-alanine amidase. Paracin 1.7 was sensitive to protease K, had antimicrobial activities at a broad pH range (3.0–8.0), and was heat resistant (121 °C for 20 min). Paracin 1.7 from *Lactobacillus paracasei* HD1-7 is a novel bacteriocin that has potential applications in food preservation.

Bacteriocins are ribosomally synthesized low-molecular weight peptides or proteins with potential use in food preservation due to their bactericidal effects on food spoilage and pathogenic organisms^1^. Based on their primary structure, molecular mass, heat stability, and molecular organization, The LAB peptide bacteriocins are divided into three classes. Class-I consists of bacteriocins (often referred to as lantibiotics) that contain one or more of the modified amino acid residues lanthionine, b-methyllanthionine, dehydroalanine, dehydrobutyrine, and/or D-alanine^2^. Class-II consists of bacteriocins that lack modified residues^2^,^3^,^4^,^5^,^6^, and include bacteriocins produced by *Lactobacillus rhamnosus* and bacteriocin Paracin 1.7 produced by *L. paracasei* HD1-7. The class-II bacteriocins are further divided into four subclasses, class- IIa, -IIb, -IIc, and -IId^2^. Class-IIa contains the antilisterial one-peptide pediocin-like bacteriocins that have similar amino acid sequences^2^,^3^,^4^,^5^, class-IIb contains the two-peptide bacteriocins, class-IIc consists of the cyclic bacteriocins whose N- and C-termini are covalently linked, and class-IId contains the one peptide non-cyclic bacteriocins that show no sequence similarity to the pediocin-like bacteriocins. Class III consists of a few large heat-labile protein bacteriocins.

Besides bacteriocins, LAB produce many other inhibitory compounds, such as organic acids, free fatty acids, ammonia, diacetyl, hydrogen peroxide, and enzymes^7^. However, bacteriocins have unique applications in food processing and food safety because of their heat stability and sensitivity to proteolytic enzymes. Nisin, a bacteriocin synthesized by *L. lactis*, has been used in several countries to extend the shelf life of food products^8^.

In previous studies, we isolated *L. paracasei* HD1-7 from Chinese sauerkraut juice^9^. Studies have assessed the optimum fermentation medium for *L. paracasei* HD1-7 growth and Paracin 1.7 synthesis^10^. Paracin 1.7, which has a wide antibacterial spectrum, has growth-inhibitory effects against Gram positive (e.g., *Staphylococcus*, *Micrococcus, Bacillus*, and *Lactobacillus*) and Gram negative bacteria (e.g., *Proteus*, *Escherichia*, *Enterobacter*, *Pseudomonas*, and *Salmonella*). This study purified and characterized Paracin 1.7 from *L. paracasei* HD1-7. The amino acid analysis of this bacteriocin will provide assistance for gene cloning and expression.

## Results

### Paracin 1.7 purification

**Figure 1:**
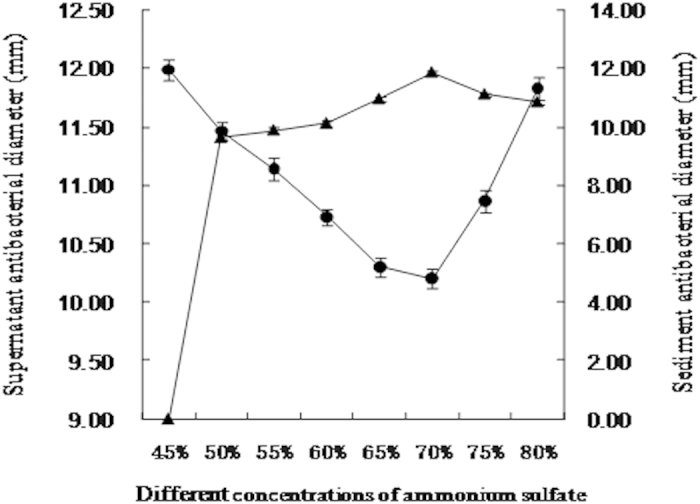
Antibacterial activities of precipitate and supernatant with different saturation of ammonium sulfate. (▲) zone of inhibition by precipitate (mm); (●) zone of inhibition by supernatant (mm).

Following ammonium sulfate precipitation, the antibacterial activities of the supernatant and precipitate were assessed (Fig. 1). There was no antibacterial activity in the precipitate following precipitation with 45% ammonium sulfate. With increasing ammonium sulfate saturation, the diameter of the zone of inhibition reached a maximum of 11.87 mm at 70% ammonium sulfate. With increasing saturation, antibacterial activity progressively decreased. Therefore, 70% ammonium sulfate was used in subsequent experiments.

The bacteriocin was further purified by CM Sepharose Fast Flow . There was one peak, which was assessed for antibacterial activity. After cation-exchange chromatography, the specific activity of the bacteriocin increased to 457 AU/ml and the final yield of the bacteriocin was 88% of the original yield. The purification fold was 1.32. The active fraction collected from CM Sepharose Fast Flow was further purified by Sephadex G-10 and tested for antibacterial activity. The specific activity of the bacteriocin increased to 365 AU/ml and the final yield was 40% of the original. The purification fold was 2.87.

The antibacterial activity, yield, fold purification, and purification method of Paracin 1.7 are summarized in Table 1.

### Molecular weight determination

Purified Paracin1.7 was extracted using a protein extraction kit (Cat#P1225, China) and analyzed by tricine-SDS-PAGE, which yielded a single band at about 10 kDa.

### Liquid chromatography/LTQ mass spectrometry and N-terminal amino acid sequencing

The N-terminal amino acid sequence was VSNTFFA. This partial sequence of Paracin 1.7 was compared to those present in the database. However, there were no significant similarities, revealing that Paracin 1.7 is a novel peptide.

The liquid chromatography/LTQ mass spectrometry results revealed that the partial sequences were homologous with those of the ABC-type oligopeptide transport system and N-acetylmuramoyl-L-alanine amidase. ABC-transporter is a specific protein, which belongs to class II bacteriocins; N-acetylmuramoyl-L-alanine amidase destroys microbial cell walls leading to cell rupture and death.

### Preliminary characterization of Paracin 1.7

The physicochemical characteristics of Paracin 1.7 were determined. The results revealed that the antibacterial activity of Paracin 1.7 was affected by temperature, pH, hydrolytic enzymes, and organic solvents (Table 2).

The antibacterial activity of Paracin 1.7 was not significantly affected following heat treatment at 40, 50, 60, 70, or 100 °C for 20 min. The inhibitory activity of bacteriocin was 82.48% of its original activity at 121 °C after 20 min. These results suggest that the bacteriocin produced by *L. paracasei* HD1-7 is resistant to heat.

At pH 3–6, the antibacterial activity of the bacteriocin was higher than that of the control. Additionally, the antibacterial activity was the highest at pH 3, decreased at pH 7–8, and disappeared at pH 9. These results suggest that the bacteriocin is affected by pH.

The effects of a number of enzymes (protease K, trypsin, α-amylase, and papainase) on bactericin were evaluated. Following treatment with them at their optimal temperature and pH, the antibacterial activity decreased. The bacteriocin from *L. paracasei* HD1-7 was sensitive to protease K and trypsin, moderately sensitive to papainase and insensitive to α-amylase. Therefore, the characteristic of the bacteriocin produced by *L. paracasei* HD1-7 are those of a peptide.

The effects of organic solvents on bacteriocin activity were assessed. Treatment with n-butanol did not affect the inhibitory activity of bacteriocin. The bacteriocin activity retained >97% when treated with methanol, ethanol, acetone, and benzene.

## Discussion

Bacteria can produce two different types of antimicrobial peptides: 1) ribosomally synthesized peptides or bacteriocins, which exhibit a relatively narrow range of antimicrobial activity, i.e., inhibit closely related bacteria and 2) non-ribosomally synthesized peptides, which exhibit broader antimicrobial activity. These antimicrobial peptides, however, have no bactericidal effects on bacteriocin-producing bacteria^1^,^11^.

In this study, bacteriocins synthesized by *L. paracasei* HD1-7 were purified by a three-step method. Following partial purification with 70% ammonium sulfate, CM Sepharose Fast Flow, and Sephadex G-10, the final yield of Paracin 1.7 was 39.93% with an specific activity and fold purification of 1.56 × 10^3^ AU/mg and 2.87, respectively. Based on the Tri-glycine-SDS-PAGE results, the molecular weight of Paracin 1.7 was about 10 kDa. The purification method used in this study was fast and easy to perform.

The N-terminal amino acid sequence of Paracin 1.7 was VSNTFFA (Val-Ser-Asn-Thr-Phe-Phe-Ala). The enzymatic results revealed that Paracin 1.7 was sensitive to protease K and trypsin, and relatively sensitive to α-amylase and papainase, suggesting that the characteristic of the bacteriocin produced by *L. paracasei* HD1-7 are those of a peptide.

The LC\LTQ results revealed that the bacteriocin contains an ABC-transporter protein that belongs to class II bacteriocins. The ABC-transporter protein is an ATP enzyme which is anchored to the bacterial membrane. It can catalyze the flip of lipid in the bilayer and change their conformation. N-acetylmuramoyl-L-alanine amidase binds to the ABC-transporter protein and is transferred into the cell, affecting synthesis and transport of macromolecules and contributing to cell lysis and apoptosis^5^. This protein protects cells from being killed by self-generated bacteriocins^6^.

The bacteriocin Paracin 1.7 does not contain YGNGVXC, which is specific to class IIa. This sequence can promote non-specific binding between bacteriocins and the target cell surface. This sequence may penetrate the helical structure of the cytomembrane, change the permeability of mannose phosphate molecules embedded in the membrane, cause conformational changes to the protein, and contribute to membrane-leakage and cell death^12^,^13^. Furthermore, the inhibitory effect of Paracin 1.7 did not depend on the YGNGVXC sequence^14^. Class IIb bacteriocins consist of two different peptides whose genes are next to each other in the same operon. All two-peptide bacteriocins identified to date contain GxxxG-motifs (x representing any sequence)^15^. The trans-membrane helix–helix structure of class IIb might interact with an integrated membrane protein, but a membrane-associated receptor for a two-peptide bacteriocin has yet to be identified^15^. Paracin 1.7 does not contain a GxxxG motif and it had only one activate peak on HPLC chromatography and one band by SDS-PAGE. Therefore it does not consist of two peptides, as would be expected if it belonged to Class IIb. To determine the class of Paracin1.7, the bacteriocin was treated with dithiothreitol (DTT). Class IIc bacteriocins can be activated by thiols^16^ The results revealed that bacteriocin retained its antibacterial activity and did not migrate in tricine-SDS-PAGE (data not shown); therefore, Paracin 1.7 does not belong to class IIc.

Based on comparisons of the N-terminal sequence, there were no bacteriocins similar to Paracin 1.7. While the N-terminal sequence does not contain cationic amino acids, such as lysine or arginine, it still has antibacterial activity. Therefore, Paracin 1.7 might be similar to the bacteriocin reported by Sebei^17^; it does not have cationic amino acids and possesses a strong antibacterial activity. Based on the results, Paracin 1.7 may belong to class IId^18^.

Paracin 1.7 was stable to heat treatment at 40–70 °C for 20 min. Following heat treatment at 121 °C for 20 min, Paracin 1.7 retained 82% of its original antibacterial activity. Following storage 4 °C for six months, there was no loss in antibacterial activity of Paracin 1.7 (data not shown); therefore, this bacteriocin has broad applications in the food processing industry. The antibacterial activity of Paracin 1.7 was stable at pH 3–8. Paracin 1.7 was more stable in acidic conditions and inactivated in alkaline conditions. In contrast, nisin exhibits a strong antibacterial activity at low pH and is inactivated at pH close to 7^19^ Therefore, both nisin and paracin 1.7 are most active in acidic conditions.

Paracin 1.7 was degraded by trypsin and proteinase K, moderately sensitive to papainase and insensitive to amylase. Studies have reported that mesenterocin E131 is completely inactivated by proteinase K, trypsin, and chymotrypsin^20^; leuconocin S and carnocin 54 synthesized by *Leuconostoc paramesenteroides* are not sensitive to protease but are sensitive to amylase^21^. Bacteriocins synthesized by LAB are degraded by proteases, which prevent their accumulation in the body and contribute to drug resistance. However, bacteriocins have different sensitivities to various enzymes.

With the addition of organic solvents, large-scale production of Paracin 1.7 dry powder can be produced. The effects of organic solvents on bacteriocin activity was investigated. The results revealed that organic solvents had a relatively weak effect on bacteriocin activity; Paracin 1.7 retained >97% of its original antibacterial activity following treatment with ethanol, acetone, benzene, n-butanol, and methanol. Experiments currently undergoing in our laboratory are focusing on the applications of Paracin 1.7 in food processing technologies.

## Methods

### Strain and culture conditions

The bacteriocin-producing strain *L. paracasei* HD1-7 (CCTCCM205015), isolated from Chinese sauerkraut juice, was grown in MRS broth (Aoboxing, Beijing, China) at pH 5.5: soya peptone, 10 g/L; beef extract, 10 g/L; yeast extract, 5 g/L; glucose, 20 g/L; K_2_HPO_4_, 2 g/L; Na_2_SO_3_, 0.1 g/L; sodium acetate, 5 g/L; MgSO_4_.7H_2_O, 0.2 g/L; MnSO_4_, 0.05 g/L; ammonium citrate, 0.4 g/L; and Tween 80, 1 ml. *B. subtilis* (ATCC 11774) was used as the indicator strain in antimicrobial activity assays. The strain was grown in LB broth (Aoboxing) at pH 7.0: peptone, 10 g/L; yeast extract, 5 g/L; and NaCl, 10 g/L. Both media were autoclaved at 0.122 MPa for 15 min. For long time storage, the strains were stored at −80 °C in MRS or BP broth (beef extract, 3 g/L; peptone, 10 g/L; and NaCl, 5 g/L; pH 7.0–7.2) supplemented with 20% (v/v) glycerol, and propagated three times in MRS or BP broth prior to use. For antimicrobial activity assays, we used BP broth supplemented with 0.75% (w/v) agar for the upper medium and water agar supplemented with 2% (w/v) agar for the lower medium.

*L. paracasei* HD1-7 was grown in sterile MRS broth and incubated at 30 °C under continuous shaking (180 rpm) for 18 h until the cell density reached 10^7^ CFU/ml. The bacterial suspension was used to inoculate fresh, sterile MRS broth at 5% (v/v) and incubated for 24 h.

### Preparation of crude bacteriocin and antibacterial activity assay of Paracin 1.7

Cell free supernatant (CFS) of *L. paracasei* HD1-7 was obtained following centrifugation (Beckman, American) at 4,500 rpm for 15 min at 4 °C. CFS was further filtered with an air pump (0.2-mm pore size water filter) and concentrated in a rotary evaporator (EYELA, N1001, Japan) to 1/10^th^ its original volume.

The antimicrobial activity of Paracin 1.7 was assessed by an agar-well diffusion method^22^. The concentration of *B. subtilis* was 10^7^ CFU/ml. Antimicrobial activity was determined by measuring the diameter of the zone of inhibition around the wells and it was expressed as arbitrary units (AU) per ml. To eliminate the antimicrobial effect of lactic acid, the pH of the supernatants was adjusted to 5.5 with 1 M NaOH. Titers were defined as the reciprocal of the highest dilution that inhibited the growth of the indicator strain. Bacteriocin activity was expressed as AU/ml^9^. Protein concentrations were determined by the Bradford assay (595 nm) using bovine serum albumin as standard.

### Paracin 1.7 purification

Bacteriocin was purified by CM Sepharose Fast Flow, Sephadex, and HPLC. CFS of *L. paracasei* HD1-7 (30 ml) was loaded at 3 mL/min into a CM Sepharose Fast Flow cation-exchange column (16 mm × 200 mm), previously equilibrated with 0.05 mol/L ammonium acetate buffer (pH 6.0). Elution was performed using a stepwise ammonium acetate gradient (0.05–1.0 mol/L) at 1 ml/min and detected at 254 nm with a UV detector. Fractions were assessed for antimicrobial activity.

The active fractions (fraction I) obtained from cation-exchange chromatography were pooled, concentrated, and loaded into Sephadex columns (1000 mm × 10 mm) containing G50, G25, and G10 packing materials to further fractionate the active fraction. The columns were equilibrated with 0.05 mol/L ammonium acetate (pH 6.0) at 0.8 ml/min. Eluted fractions were detected at 254 nm; active fractions were concentrated by vacuum evaporation. Antimicrobial activity was assayed using the agar-well diffusion method. Concentrated active fractions obtained from Sephadex G10 were further purified in a Waters HPLC system (Waters 510 HPLC pump, Waters TM996 Photodiode Array Detector) equipped with a Delta-Pak TM300 C18 column (5 μm, 3.9 mm × 150 mm); the flow rate was 0.8 ml/min. Elution was performed with a gradient from 100% buffer A (5% v/v, acetonitrile/distilled water) to 100% buffer B (80% v/v, acetonitrile/distilled water) in 30 min.

First, supernatant of *L. paracasei* HD 1–7 was mixed with ammonium sulfate, resulting in different concentrations (45, 50, 55, 60, 65, 70, 75, and 80% w/v) at 4 °C under constant stirring overnight. The cell suspensions were subsequently centrifuged at 10,000 RPM for 15 min (4 °C); both the supernatant and precipitate were harvested and bacteriocin activity was measured.

The solution obtained following ammonium sulfate precipitation was loaded into a CM Sepharose Fast Flow (16 mm × 200 mm, GE HEALTHCARE, Sweden) cation-exchange column equilibrated with ammonium acetate buffer (pH 6.0). Active fractions (partially purified Paracin 1.7) were eluted using an ammonium acetate buffer gradient (0.05–1 mol/L) at 1.3 ml/min. Finally, partially purified Paracin 1.7 from CM Sepharose was purified by Sephadex G-10 (Sigma, USA) chromatography (12 mm × 200 mm) using an AKTA purifier 100 equilibrated with the same buffer. Elution was performed with ammonium acetate (pH 6.0) at 1 ml/min. Bacteriocin activity and protein concentration were measured at each step of the purification process. All experiments were conducted in triplicate.

### Molecular weight determination

Purity and molecular weight of the active fractions obtained from Sephadex G-10 were determined by tricine-sodium dodecyl sulfate polyacrylamide gel electrophoresis (SDS-PAGE)^23^ using a vertical slab gel apparatus (BG-verMINI, China) with 4% stacking and 16.5% separating gels. Bacteriocin preparations and molecular weight markers (PageRuler, Lithuania) were run at 30 mA. Following electrophoresis, the gel was cut into two parts. Half of the gel was stained with Coomassie brilliant blue G-250 (SRL, India) and de-stained in 30% (v/v) methanol/10% (v/v) glacial acetic acid until the bands were clear; the molecular weight of Paracin 1.7 was calculated from the relative mobility of the molecular weight markers. The other half of the gel was rinsed overnight in sterile water. The lanes of the gel were cut and each lane was overlaid with nutrient soft agar inoculated with indicator strain and subsequently cultured at 37 °C for 14 h^24^,^25^.

### Liquid chromatography/LTQ mass spectrometry and N-terminal amino acid sequencing

Following tricine-SDS-PAGE, the protein of interest was excised from the gel. An LTQ linear ion trap mass spectrometry (ThermoFinnigan, USA) coupled to an Agilent 1100 HPLC system was used for amino acid sequencing. The results were compared with a database. Partial sequencing was determined according to the spectral library.

The bacteriocin was electrophoretically transferred to a 0.2-μm pore size PVDF membrane^26^. N-terminal sequencing of bacteriocin was performed by GeneCore BioTechnologies (China) using dry PVDF membranes, which contained the bacteriocin band. Edman degradation analysis was performed in a Procise 492cLC Protein Sequenator (Applied Biosystems, USA) to determine the first seven amino acids in bacteriocin. The amino acid sequence was compared to those of known bacteriocins at http://aps.unmc.edu/AP/main.php.

### Preliminary characterization of Paracin 1.7

The physicochemical characteristics of Paracin 1.7 that were evaluated included its temperature, pH, enzymatic, and organic solvent sensitivities. Additionally, the preservation life of Paracin 1.7 was determined. To determine its temperature sensitivity, Paracin 1.7 was heated to 40, 50, 60, 70, 100, and 121 °C for 20 min. Residual bacteriocin activity was measured at each temperature using unheated Paracin 1.7 as the control.

Paracin 1.7 was adjusted to pH 3, 4, 5, 6, 7, 8, and 9 using sterile 5 mol/ml NaOH or HCl and maintained at the corresponding pH for 20 min. Residual bacteriocin activity at each pH was measured using unadjusted Paracin 1.7 as the control.

The sensitivity of Paracin 1.7 to different enzymes, including protease K (Sigma, USA), trypsin (Ameresco, USA), α-amylase (Sigma, USA), and papainase (Sigma, USA), was evaluated by incubating Paracin 1.7 in the presence of each enzyme (1 mg/ml) at the optimal temperature and pH for 1 h. The mixture was subsequently heated to 100 °C for 5 min to inactivate the enzymes. Residual bacteriocin activity was measured using Paracin 1.7 without enzymatic treatment as the control.

The tolerance of bacteriocin to different organic solvents was assessed by incubating Paracin 1.7 for 1 h in the presence of 10% (w/v) organic solvents (methanol, ethanol, acetone, benzene, and n-butanol) at 25 °C. Residual bacteriocin activity was measured using Paracin 1.7 without organic solvents as control.

The preservation life of bacteriocin was assessed by storing Paracin 1.7 for 6 months at 4 °C. Residual bacteriocin activity was determined every 15 days.

Residual bacteriocin activity was assayed by the agar well diffusion method and calculated based on the ratio of inhibitory activity with treatment and inhibitory activity without treatment (i.e., control). Statistical analyses were performed by SPSS 13.0.

## Additional Information

**How to cite this article**: Ge, J. *et al*. Purification and Partial Characterization of a Novel Bacteriocin Synthesized by *Lactobacillus paracasei* HD1-7 Isolated from Chinese Sauerkraut Juice. *Sci. Rep*. **6**, 19366; doi: 10.1038/srep19366 (2016).

## Figures and Tables

**Table 1:** Purification and activity of Paracin 1.7.

Purification stage	Volume (ml)	Total protein (mg)	Total activity (AU)	Specific activity (AU/mg)	Fold Purification (per step)	Yield (%) (per step)
CFS concentration	50.00	81.20	2.73 × 10^4^	336.10	1	100.00
Ammonium sulfate precipitation	50.00	62.93	2.58 × 10^4^	410.34	1.22	94.62
CM Sepharose FF	50.00	42.20	2.28 × 10^4^	541.96	1.32	88.49
Sephadex G-10	25.00	5.87	9.12 × 10^3^	1555.50	2.87	39.93

**Table 2:** Characterization of Paracin 1.7.

Treatment	Diameter of zone of inhibition (mm)	Antibacterial activity (%)
Temperature		
40 °C, 20 min	17.34 ± 0.05b	98.30
50 °C, 20 min	17.32 ± 0.03b	98.19
60 °C, 20 min	17.23 ± 0.02b	97.68
70 °C, 20 min	17.25 ± 0.06b	97.79
100 °C, 20 min	16.83 ± 0.06c	95.41
121 °C, 20 min	14.55 ± 0.07d	82.48
Control (37 °C)	17.64 ± 0.05a	100
pH		
3	46.43 ± 0.06c	174.48
4	43.70 ± 0.07c	164.22
5	41.25 ± 0.04c	155.02
6	31.59 ± 0.02c	118.71
7	21.15 ± 0.05b	79.48
8	15.75 ± 0.06b	59.19
9	0d	
Control (6.5)	26.61 ± 0.06a	100
Enzymes		
Protease K	31.08 ± 0.11e	90.27
Trypsin	31.61 ± 0.16d	91.81
α- Amylase	31.97 ± 0.07c	92.86
Papainase	32.39 ± 0.09b	94.07
Control	34.43 ± 0.10a	100
Organic solvents		
Methanol	26.51 ± 0.04e	97.11
Ethanol	26.65 ± 0.04d	97.62
Acetone	26.70 ± 0.05d	97.80
Benzene	26.79 ± 0.02c	98.13
n-Butanol	27.09 ± 0.02b	99.23
Control	27.30 ± 0.03a	100

Same letters represent significant differences (p < 0.05) relative to the control.

Different letters represent significant differences (p > 0.05) relative to the control.
